# Assessing the medical resources in COVID-19 based on evolutionary game

**DOI:** 10.1371/journal.pone.0280067

**Published:** 2023-01-11

**Authors:** Keyu Guo, Yikang Lu, Yini Geng, Jun Lu, Lei Shi

**Affiliations:** 1 Information School, The University of Sheffield, Sheffield, United Kingdom; 2 School of Statistics and Mathematics, Yunnan University of Finance and Economics, Kunming, Yunnan, China; 3 Interdisciplinary Research Institute of Data Science, Shanghai Lixin University of Accounting and Finance, Shanghai, China; 4 MOE-LCSM, School of Mathematics and Statistics, Hunan Normal University, Changsha, China; 5 Key Laboratory of Applied Statistics and Data Science, Hunan Normal University, College of Hunan Province, Changsha, China; CNR, National Research Council of Italy, ITALY

## Abstract

COVID-19 has brought a great challenge to the medical system. A key scientific question is how to make a balance between home quarantine and staying in the hospital. To this end, we propose a game-based susceptible-exposed-asymptomatic -symptomatic- hospitalized-recovery-dead model to reveal such a situation. In this new framework, time-varying cure rate and mortality are employed and a parameter *m* is introduced to regulate the probability that individuals are willing to go to the hospital. Through extensive simulations, we find that (1) for low transmission rates (*β* < 0.2), the high value of *m* (the willingness to stay in the hospital) indicates the full use of medical resources, and thus the pandemic can be easily contained; (2) for high transmission rates (*β* > 0.2), large values of *m* lead to breakdown of the healthcare system, which will further increase the cumulative number of confirmed cases and death cases. Finally, we conduct the empirical analysis using the data from Japan and other typical countries to illustrate the proposed model and to test how our model explains reality.

## Introduction

The COVID-19 sweeping across all countries has so far left more than 430 million people infected and caused almost 6 million deaths (data from the World Health Organization). Understanding its transmission mechanism is one of the most pressing social challenges we face today. Numerous studies on this subject provide a wealth of theoretical models to mimic the spread of disease under different conditions [1–7]. However, the critical subject of COVID-19 is to not only predict disease outbreaks but also explore how to prevent pandemics.

To contain the pandemic more scientifically and effectively, physical protection [8–14], individual measures [8, 9, 15], national measures [11, 12, 16–20] and so on have been deeply explored. The effects of wearing masks [8–10], social distancing [11, 12, 15–17], lockdown [18–20] have been studied extensively. Wearing a mask is an effective preventive measure, and an evidence analysis [8] has supported mass masking in this pandemic. Therefore, many countries required their citizens to wear masks in public places [21], which blocked the transmission of the disease. Akin to wearing masks, social distancing successfully reduces the risk of infection. The results of a survey in 58 cities in China showed that the epidemic would have been more severe if social distance had been delayed by one day [15]. Likewise, lockdowns essentially stop the spread of the disease as a way of preventing people with the disease from moving around. Although such a policy would be economically devastating, it would stop the movement of infected people, thereby reducing the cost of an outbreak. The actual situation in Wuhan supports the conclusion [18]. The above measures have a common goal, that is, separating susceptible individuals from infected individuals as soon as possible. In other words, these measures reduce the contact rate between susceptible and infected individuals. A vital aspect of stopping the pandemic is ensuring that healthcare resources are allocated effectively and sufficiently. A growing body of literature on resource distribution has offered insightful advice based on the results of research [22–25]. When resources for masks are limited, prioritized coverage of the elderly is the optimal strategy, rather than a random distribution [22]. In addition, limited vaccine resource distribution is of primary importance. In almost all circumstances, reducing fatalities required distributing the vaccine to older adults who are most at risk of death [24]. Among these resources, the factors related to the medical system can not be ignored in that its capacity to prevent the spread of pandemic diseases is also crucial. If the healthcare system is broken, the epidemic would bring worse results [26]. Using linear and mixed-integer programming models, the authors found that an optimal configuration could reduce cases in New Jersey, Texas, and Miami by at least 85% [27].

The recent emergence of mutated viruses has brought a major test to humans’ fight against the epidemic and would bring a more overwhelming wave of the pandemic if some measures could not be carried out better. In such circumstances, how to make the healthcare system operate more efficiently and effectively is critical to preventing COVID-19. We propose the game-based susceptible-exposed-asymptomatic -symptomatic-hospitalized-recovery-dead (Game-based SEAIHRD) model to describe the dynamics of the epidemic, where the factor related to medical resources is involved and the human’s decision to go to the hospital is considered using the evolutionary game theory. The framework of evolutionary games provides an available tool to investigate the epidemic problem related to human behaviors [28–33]. According to the difference in human behavior [34–36], we define a parameter *m* to represent individual motivation to seek medical care. By extensive simulation, we find that given a low infectious rate, the *m* plays a positive role in containing the pandemic. In other words, increasing *m* makes the predicted cumulative number of confirmed cases and death decrease. However, facing a pandemic with a high transmission rate, little *m* can contain the pandemic. But a high value of *m* makes the medical system broken, making both the cumulative number of confirmed cases and death increase. In addition, we select Japan as a sample to examine the performance of the model. After obtaining parameter estimates from actual data, simulations show the amount of medical resources is 2530, which indicates the medical system can provide medical assistance to 2530 patients at one time, and the contact rates need to be controlled at the early stage of the pandemic by effective measures.

The paper is organized as follows: We introduce the dynamics of the game-based SEAIHRD model in Section 2. The technical realization of the method and analytic results are shown in Section 3. The conclusion and discussion about this model are given in Section 4. Some detailed materials including the algorithm are listed in the additional information section.

## Materials and methods

In this section, the fundamental assumptions underlying the model are as follows: (i) Virus mutation isn’t involved; (ii) The changes in morbidity and mortality caused by individual factors in different populations are ignored; (iii) The re-infected process is ignored [37]; (iv) Modeling the early stage of COVID-19 ignores vaccinated individuals and reinoculate individual.

In this paper, we build a game-based SEAIHRD compartment model to characterize the propagation dynamics of COVID-19. In a healthcare system that has two states: normal operation and collapse, an individual has a large probability of going to the hospital when it’s running normally; otherwise, it’s little. When the healthcare system is at normal running, the patient can get effective treatment. The healthcare system being collapse means the existing medical system and medical resources cannot meet the needs of patients and can not cope with the outbreak, and a state that is far beyond the saturation or tolerance limit present, thus causing the epidemic to be out of control. Thus, we introduce a parameter *m* to represent individual motivation to seek medical care. This parameter can display the difference to seek medical care in different regions.

This system is partitioned into seven exclusive stages: susceptible (S), exposed (E), asymptomatic (A), symptomatic (I), hospitalized (H), recovered (R), and dead (D). Susceptible individuals who are not infected by the disease can be infected by infected individuals, namely, asymptomatic individuals and symptomatic individuals, in many ways. However, they go through an incubation period before they become infected individuals. Individuals in this period are known as exposed individuals. Although they already have the virus in their bodies, the viruses do not come into play and they can not pass it to other susceptible individuals. After this period, infected individuals can be divided into asymptomatic individuals and symptomatic individuals according to whether their bodies are accompanied by symptoms. Considering asymptomatic individuals are harder to detect unless they have nucleic acid detection, we assume that asymptomatic individuals do not go to the hospital voluntarily, thus the difference between asymptomatic and symptomatic individuals is that only symptomatic individuals have a choice to go to the hospital for treatment.

**Fig 1:**
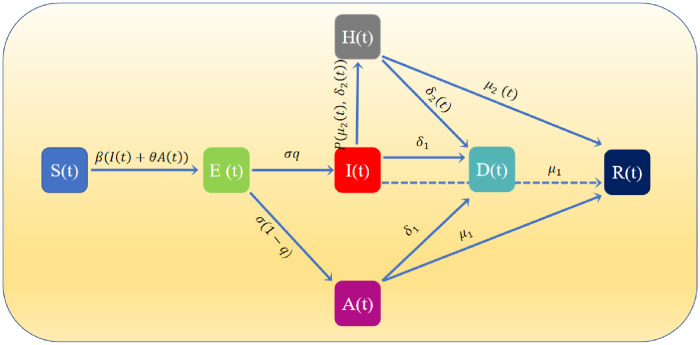
Graphical scheme representing the interactions among different stages of infection in the mathematical model.

The interactions among these healthy statuses are shown in Fig 1. More specifically, susceptible individuals can be infected and become exposed individuals when they encounter infected individuals with the probability *ϵ*. The transmission rates are *β* and *θβ*, respectively, when infected by symptomatic and asymptomatic individuals. After the incubation period of 1/*σ* days, *q* of the exposed individuals become symptomatic individuals, and the rest turn into asymptomatic individuals. Since the cure rate is lower and the death rate is higher when the healthcare system collapses. Thus, for symptomatic individuals, whether to go to the hospital depends on the Fermi function. That is, *P*(*μ*_2_(*t*), *δ*_2_(*t*)) represents the probability that symptomatic individuals go to the hospital.

$$\begin{array}{c} P\left(\mu_{2}\left(t\right),\delta_{2}\left(t\right)\right)=\frac{m}{1+exp(\left(\delta_{2}\left(t\right)-\mu_{2}\left(t\right)\right)/K)}, \end{array}$$ (1)

where *μ*_2_(*t*) and *δ*_2_(*t*) measure the cure rate and the death rate, which also determine the probability of hospitalized individuals transforming into recovered individuals and death individuals. K is the amplitude of noise or its inverse ratio is the so-called selection intensity, generally *K* = 0.1 [38, 39]. Hospitalization is more likely to be chosen when the cure rate exceeds the death rate in the hospital. According to surveys from different countries [40], we introduce the regulatory factor of individual will *m* to regulate the probability that individuals go to the hospital. A large *m* indicates that individuals are more likely to the hospital.

The mathematical model describing the above process is given in Eq (2). The two time-varying factors *μ*_2_(*t*) and *δ*_2_(*t*) are dependent on the number of hospitalized individuals *H*(*t*) and the level of medical resources *α* in the healthcare system. For example, the cure rate is high and the death rate is low when medical resources are adequate. There are several research proposed a specific functional model for modeling infectious rates and cure rate, and using real data to estimate the involved parameters [41, 42], however, our model here only uses its dependence on *H*(*t*) and *α* to characterize their relationship and estimate them through the model, which is more flexible.

In Eq (2), *s* and *r* regulate the sensitivity change of the cure rate and death rate, respectively. Note that *μ*_2_(*t*) and *δ*_2_(*t*) have range where the value of *μ*_2_(*t*) can be replaced by *μ*_*min*_ (*μ*_*max*_) when the cure rate is less (greater) than the given minimum (maximum) value, and so is *δ*_2_(*t*). Symptomatic individuals who failed to get to the hospital either recover by themselves with *μ*_1_, or die with the probability *δ*_1_. Since asymptomatic individuals have no chance to go to the hospital, they can recover and die with the same probability as symptomatic individuals. Note that N in Eq (2) is the effective population (total population multiplied by *ϵ*). For convenience, we compile all the parameters and their explanations in Table 1 and formulate the model in Eq (2).

$$\begin{array}{c} \left\{ \begin{array}{c} \frac{dS(t)}{dt}=-\beta S\left(t\right)\left(I\left(t\right)+\theta A\left(t\right)\right)/N\\ \;\\ \frac{dE(t)}{dt}=\beta S\left(t\right)\left(I\left(t\right)+\theta A\left(t\right)\right)/N-\sigma E\left(t\right)\\ \;\\ \frac{dI(t)}{dt}=\sigma qE\left(t\right)-\mu_{1}I\left(t\right)-\delta_{1}I\left(t\right)-P\left(\mu_{2}\left(t\right),\delta_{2}\left(t\right)\right)I\left(t\right)\\ \;\\ \frac{dA(t)}{dt}=\sigma \left(1-q\right)E\left(t\right)-\mu_{1}A\left(t\right)-\delta_{1}A\left(t\right)\\ \;\\ \frac{dH(t)}{dt}=P\left(\mu_{2}\left(t\right),\delta_{2}\left(t\right)\right)I\left(t\right)-\delta_{2}\left(t\right)H\left(t\right)-\mu_{2}\left(t\right)H\left(t\right)\\ \;\\ \frac{dD(t)}{dt}=\delta_{1}\left(I\left(t\right)+A\left(t\right)\right)+\delta_{2}\left(t\right)H\left(t\right)\\ \;\\ \frac{dR(t)}{dt}=\mu_{1}\left(I\left(t\right)+A\left(t\right)\right)+\mu_{2}\left(t\right)H\left(t\right)\\ \;\\ \frac{d\mu_{2}\left(t\right)}{dt}=s\left(\alpha -H\left(t\right)\right)\\ \;\\ \frac{d\delta_{2}\left(t\right)}{dt}=r\left(H\left(t\right)-\alpha \right) \end{array}\right. \end{array}$$ (2)

**Table 1:** The parameter explanation in the model.

Symbol	Description
*S*(*t*)	the fraction of susceptible individuals
*E*(*t*)	the fraction of exposed individuals
*I*(*t*)	the fraction of symptomatic individuals
*A*(*t*)	the fraction of asymptomatic individuals
*H*(*t*)	the fraction of confirmed individuals that to be tested and to be sent to the hospital
*D*(*t*)	the fraction of dead individuals
*R*(*t*)	the fraction of recovered individuals
*ϵ*	the contact rate among infected individuals and susceptible individuals
*β*	infection rate of symptomatic infected
*θ*	the ratio of infection rate of symptomatic infected and infection rate of asymptomatic infected
*σ*	incubation rate for the transition from exposed to infected
*m*	regulatory factor of individual will
*δ*_1_	death rate (from infected individuals)
*μ*_1_	cure rate (from infected individuals)
*δ*_2_(*t*)	death rate (from individuals in the hospital)
*μ*_2_(*t*)	cure rate (from individuals in the hospital)
*δ*_*max*_	the maximum death rate (from individuals in the hospital)
*δ*_*min*_	the minimum of death rate (from individuals in the hospital)
*μ*_*max*_	the maximum cure rate (from individuals in the hospital)
*μ*_*min*_	the minimum of cure rate (from individuals in the hospital)
*q*	the ratio of symptomatic infection
*α*	the hospital resource level
*r*	regulatory factor of death rate
*s*	regulatory factor of cure rate

This model is a bilinear system with nine differential equations. Here, the system is positive, that is to say, the fraction of all the states and rates are non-negative values. To meet the mass conservation property of the system, an important rule is that the sum of change from all states (i.e. sum of the first seven terms on the left side of Eq (2)) equals zeros since

S(t)+E(t)+I(t)+A(t)+H(t)+D(t)+R(t)=N.

In Eq 2, the death rate and cure rate are dependent on time *t* which can well reflect the real situations.

For further analysis, we solve the basic reproductive number of an infection of the system. The basic reproductive number of an infection is the expected number of cases directly generated by one case in a population where all individuals are susceptible to infection. To calculate it, we adopt the next generation matrix approach [46, 47]. By using the notations as [47], it follows that the matrices F of new infection terms and V of the remaining transfer terms associated with the model are given:

$$\begin{array}{c} F=[\begin{array}{ccc} 0 & \beta & \theta \beta \\ 0 & 0 & 0\\ 0 & 0 & 0 \end{array}] \end{array}$$ (3)

$$\begin{array}{c} V=[\begin{array}{ccc} \sigma & 0 & 0\\ -\sigma q_{a} & \delta_{1}+\mu_{1}+P\left(\mu_{2},\delta_{2}\right) & 0\\ -\sigma (1-q) & 0 & \delta_{1}+\mu_{1} \end{array}] \end{array}$$ (4)

By solving the maximum eigenvalue of $FV^{-1}$, the basic reproduction number of the model marked by $R_{0}$ is given by

$$\begin{array}{c} R_{0}=\frac{\beta q}{\delta_{1}+\mu_{1}+P\left(\mu_{2},\delta_{2}\right)}-\frac{\beta \theta (q-1)}{\delta_{1}+\mu_{1}} \end{array}$$ (5)

## Results

### 0.1 Simulation results

**Fig 2:**
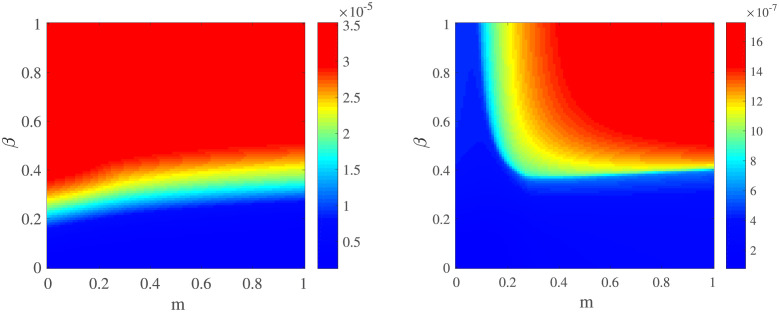
The predicted cumulative number of confirmed cases (left) and death (right) as a function of the regulatory factor of individuals will *m* and *β*. Other parameters are shown in Table 2.

In Fig 2, we provide the predicted cumulative number of confirmed cases (left) and deaths (right) which is the function of *m* and *β*. Experience tells us that a pandemic with a low infectious rate can be controlled easily, which can be verified by Fig 2. The medical system remains stable and in the normal range, even at high values of *m*, when *β* is low. A higher value in *m* predicts a lower the predicted cumulative number of confirmed cases and deaths. These phenomena indicate that *m* mitigates pandemic outbreaks given a less rate of transmission. The impact of *m* can be double-edged when infection rates are high. On the one hand, lower levels of it can contain outbreaks and keep the cumulative number of deaths low. On the other hand, high values in *m* can, however, cause an increase in confirmed cases and deaths due to a broken medical system.

**Table 2:** Parameter in the model.

Symbol	Value
*N*	200000000
*μ*_*max*_	0.3
*μ*_*min*_	0.05
*μ*_1_	0.096 [43]
*δ*_*max*_	0.02
*δ*_*min*_	0.005
*δ*_1_	0.00625 [43]
*α*	0.01
*β*	1.02 [43]
q	0.18 [44]
m	0.31
r	0.2
s	0.3
*ϵ*	0.000056 [45]
*σ*	1/7 [45]
*θ*	0.447 [44]

**Fig 3:**
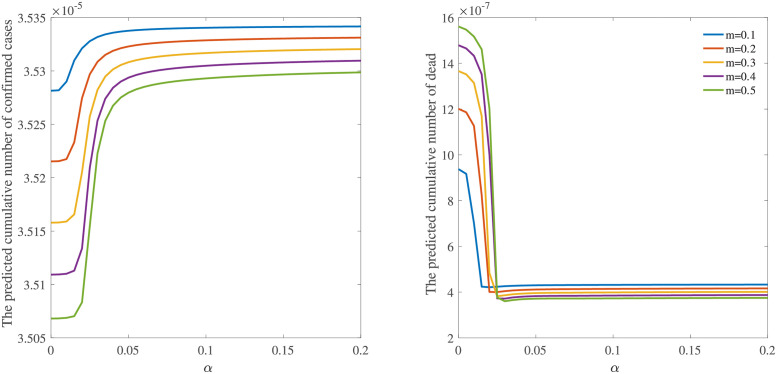
The predicted cumulative number of confirmed cases (left) and death (right) as a function of the hospital resource level *α* for different *m*. Other parameters are shown in Table 2.

We study how factors related to medical resources affect the diseases results in Fig 3. The critical factors in the model are *m* and *α*. A high *m* indicates that infected individuals prefer to go to the hospital. *α* represents the medical resource level. When the total number of infections is constant, the increment in *α* pushes the cumulative number of confirmed cases to higher values until it reaches a stable value and makes the cumulative number of death cases reach a low value, suggesting adequate medical treatment can reduce death rates. While, once the medical resource is above a certain level, it does not work. In other words, even given *α* a higher value, the cumulative number of confirmed cases and death stay at certain values. These results can be easily understood if we notice that increasing proportions from the infected individuals to patients in the hospital is a constant value. The fraction of patients depends on *m*. Under the normal operating condition of the medical system, improving the value of *m* always lowers the cumulative number of confirmed cases and death, indicating that a higher of *m* facilitates the control of the pandemic.

**Fig 4:**
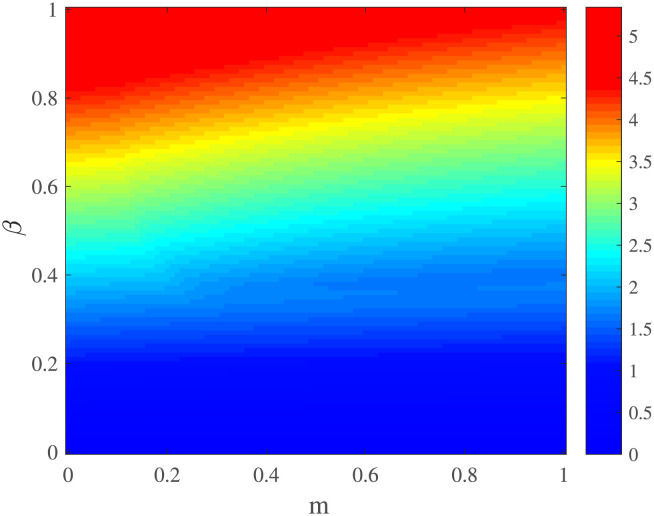
The effect of behavior on the basic reproductive number. The basic reproductive number $R_{0}$ as function of regulatory factor of hospital resource level *m* and *β*. Other parameters are shown in Table 2.

In Fig 4, we plotted the basic reproductive number $R_{0}$ for different values of *β* and *m*. Reasonably, for all values of *m*, the $R_{0}$ grows with the increase in virus transmissibility. *m* also plays an inhibitory role in the spreading dynamics. In other words, *m* support a low epidemic size and thus also a smaller $R_{0}$.

### 0.2 Real example

**Fig 5:**
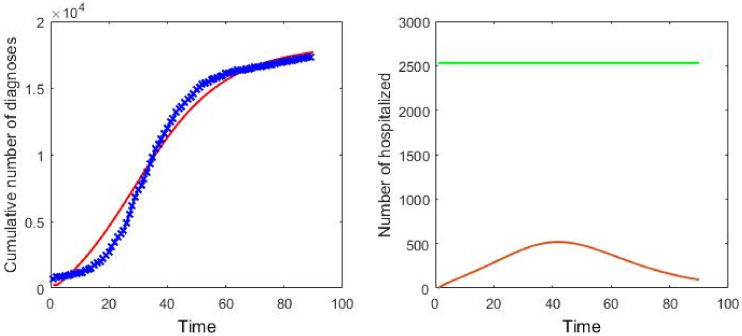
In the left panel, the cumulative number of confirmed cases in Japan. The solid lines are obtained from the theoretical model, and the dots correspond to the real cases. In the right panel, the evolution of the in-patient number *H* (the bottom line) and medical resource capacity *αN* (the top line). The parameters in this model are selected from the fitting results in Table 3.

As examples of the performance of the model, we examine the early stage of the pandemic in eight countries (the United States of America, Brazil, Britain, China, Germany, Italy, Japan, and Spanish). In these countries, the medical resource level presents an optimal value in Japan. Thus, in this section, we select Japan as an example to verify our model. The parameters in Table 1 are estimated by minimizing the mean square error between predicted values from the model (2) and real observations and solved by grid search algorithm [48]. The estimators of parameters are given in Table 3, where we only selected the data in the early 90 days after the outbreak since our purpose is to study the maintenance of medical resources in the early stage of the outbreak of COVID-19. The details of data collection and algorithm are given in additional information. At the initialized time, only infected individuals and susceptible show in the system, and the initial number of infected individuals are the same as the initial infected cases reported by governments (the time that cases are reported by governments is listed in Table 3). In the left panel of Fig 5, the blue points represent realistic data and the red line represents the fitted data. As shown in the left panel of Fig 5, our model can fit the data very well on the cumulative number of diagnoses.

**Table 3:** Parameter estimation.

Symbol	Fermi function	conformity-driven	No behavioral
*μ*_*max*_	0.16	0.3	0.3
*μ*_*min*_	0.1	0.005	0.1
*μ*_1_	0.1	0.01	0.01
*δ*_*max*_	0.025	0.06	0.06
*δ*_*min*_	0.01	0.005	0.0015
*δ*_1_	0.02	0.00625	0.00625
*α*	0.01	0.01	0.01
*β*	1	1	1
q	0.18	0.18	0.18
m	0.31	0.53	-
r	1.4e-3	0.2	1.4e-3
s	0.3	0.3	0.3
*ϵ*	1.9e-4	1.38e-4	1.3e-4
*σ*	0.6	0.6	0.6
*θ*	1e-5	1e-5	1e-5
Initial Data	Jan. 6th	Jan. 6th	Jan. 6th

Furthermore, we look at the epidemic severity and estimated capacity of medical resources. Medical resource capacity is given by *αN* in our model which is the maximum number of people that can be accommodated by the hospital. We plot *αN* and hospitalized number with the time in the right panel of Fig 5 to show the number of medical resources. For convenience, we set the days that the first case was reported by the government as day 1. Except for the cumulative number of confirmed cases that can be regarded as a general measure of the epidemic severity, the days, in-patient number exceeds the local medical resources capacity, can also be an important index. In fact, Japan has its hospitalized patients (brown line) low obviously than the medical resource capacity whose value is 2530 (green line). The number of people infected is small, and the virus epidemic was not serious in the early stage of the virus outbreak.

The results of the above analyses indicate that our model accurately predicts confirmed cases in Japan. To further verify this approach, analysis for more countries is studied. These countries include the United States of America, Brazil, Germany, and Italy. S1 Table in S1 File summarizes the values of these parameters. In S1 Fig in S1 File, the blue points represent realistic data and the red line represents the fitted data. As shown in S1 Fig in S1 File, our model can fit the data very well on the cumulative number of diagnoses and the results of the experiment show that the proposed approach has good robustness. In this section, we consider only the Fermi function as a decision function. We also present results based on conformity-driven update rules and rules without behavioral aspects. Values of the estimated parameter can be seen in Table 3. These results indicate that high consistency between predicted cases and real data, see S2 Fig in S1 File. See S1 File (Robust Analysis) for details.

**Fig 6:**
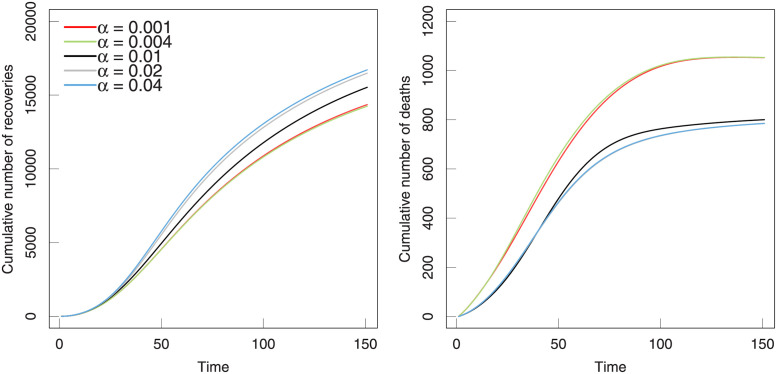
The predicted cumulative number of recovery and death in dependence on the hospital resource level *α* over time in Japan. The real situation replaced by the parameters fitted by the model (shown in Table 3) are marked with solid black lines. Note that the curves of predicted cumulative numbers of recovery are overlapped or indistinguishable for some different *α*.

In Fig 6, we study the impact of the hospital resource level *α* on the pandemic of COVID-19 through simulations. Here, we vary the values of *α* but fix the other parameters at their estimated values in Table 3. Through simulations, we obtain the changes in the cumulative number of recovery and dead individuals in dependence on *α* over time in Fig 6. With the increase in medical resources, the number of recoveries increased but the number of deaths decreased, which conforms to common sense. In general, it is shown that the cumulative number of death is more sensitive than that of the cumulative number of recoveries, which implies that the medical resource level is important for decreasing the number of deaths. For the cumulative number of deaths, the situation of Japan can not be improved much even increase the medical resource levels but will cause matter if decreasing the medical resource level, where two curves with the high level (*α* = 0.02, 0.04) overlap (or almost overlap) with real case (*α* = 0.01).

**Fig 7:**
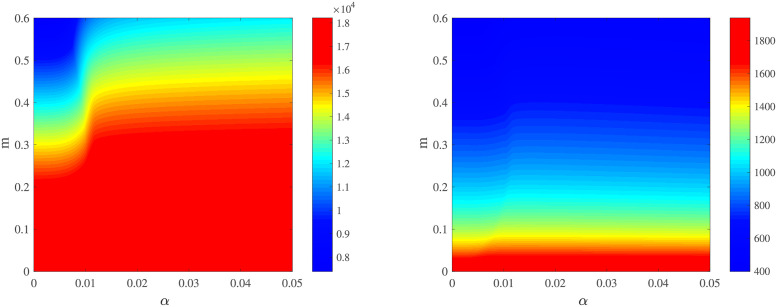
The predicted cumulative number of recovery (left) and death (right) as a function of the hospital resource level *α* and regulatory factor of individual will *m* in Japan. Other parameters are shown in Table 3.

According to the above discussion, we show that *α* has a remarkable effect on the cumulative number of recoveries and deaths, as illustrated in Fig 6. To reveal the combined effect of medical factors, we plot the cumulative number of recoveries (see left panel of Fig 7) and deaths (see right panel of Fig 7) as a function of the hospital resource level *α* and *m*. It is indicated by Fig 7 that improving *m* and *α* can ease outbreak. Whether increment occurs at *α* or *m*, it always produces extremely useful results where rates in cumulative morbidity and mortality reduce both. Whenever *α* and *m* exceed some limits, the pandemic level and the number of dead converge to zero, indicating that COVID-19 has disappeared. The one indicator (cumulative number of recoveries), experiences three-stage as *α* increasing: slow-growth, fast-growth, and second slow-growth. In the first stage, the cumulative number of recoveries increases gently as *α* increases. In the second stage, it climbs faster comparing the previous phase. At the last stage, the growth rate in the indicator becomes gentle, even nil with *α* increasing. For the lower-level regulatory factor of individuals will *m*, the toll is great. In particular, in the condition of *m* ≥ 0.1, the highest cumulative death rate shows up when *α* = 0.03.

**Fig 8:**
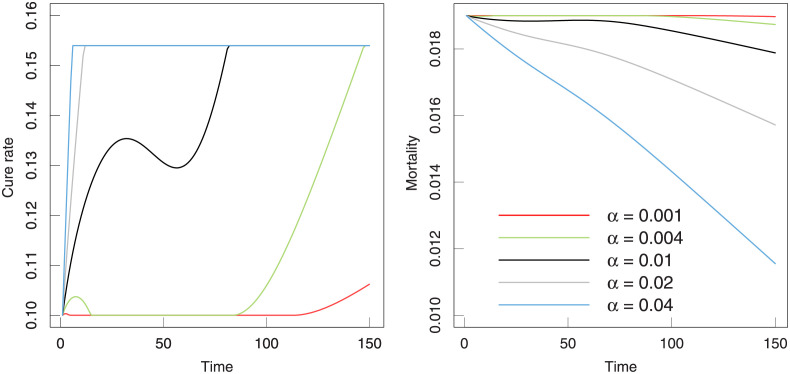
The evolution of the cure rate and mortality. The parameters in this model are selected from the fitting results in Table 3.

In Fig 8, the cure rate and mortality as a function of time are listed. The trends in two rates with time present a similar variation and shows a negative relationship. More specifically, the cure rate goes to rise and mortality falls off at the same times for different *α*. The mortality presents a stable decrease with time and the difference for various *α* is obvious.

**Fig 9:**
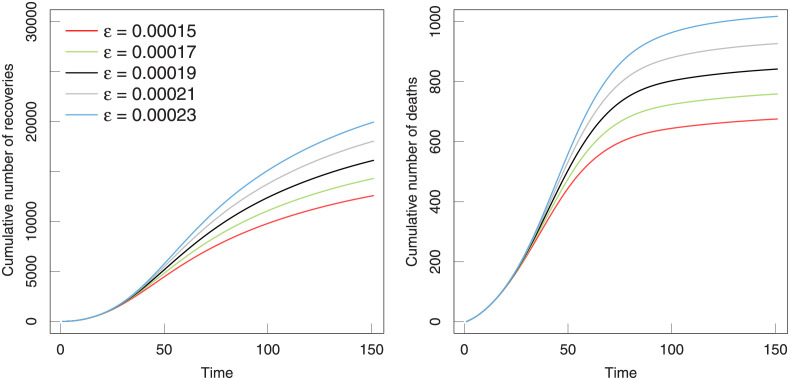
The temporal evolution of the cumulative number of recovery and dead individuals predicted in dependence on contact rate *ϵ*. The real situation replaced by the parameters fitted by the model (shown in Table 3) are marked with solid black lines.

In addition, we further consider the effect of the contact rate among infected and susceptible individuals and display the temporal evolution of the cumulative number of recovery and dead individuals in dependence on *ϵ* in Fig 9. It is clear that a high contact rate increases obviously the cumulative number of recoveries and deaths. The relatively high contact rate leads to a high cumulative recovery number because the total number of infected groups has increased at this time. The cumulative number of recoveries and the number of deaths have a trend of slowing down.

**Fig 10:**
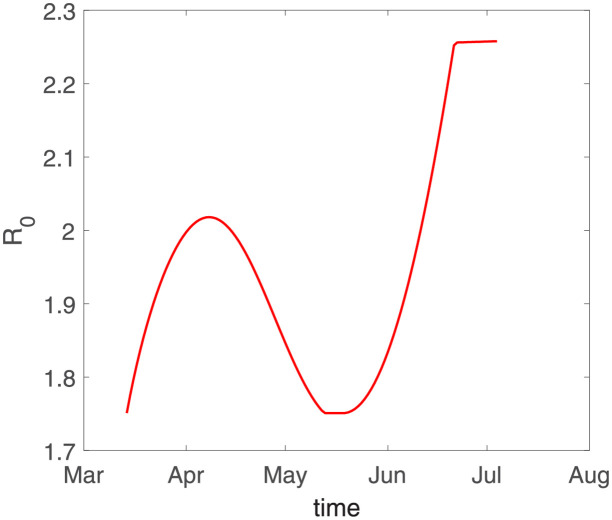
The evolution of the basic reproductive number $R_{0}$. The parameters in this model are selected from the fitting results in Table 3.

The evolution of the basic reproductive number $R_{0}$ is shown in Fig 10. Although $R_{0}$ increases again at the end of the early stage in Japan, its value does not exceed 2.3, which is also a lower level of the basic reproductive number. Thus, the epidemic in Japan is well controlled in the early stage.

## Conclusion

Previous works [49–52] have proved that medical resources appear some deficiencies when facing the pandemic, such as unfair allocation of scarce medical resources [50], medical resource deficiency [53], and so on, which will seriously reduce the efficiency of epidemic prevention. In this work, we propose the game-based SEAIHRD model with adjustable hospitalization rates to describe the dynamics of COVID-19. This model accounts for the effects of medical resource and incorporate the strategy of the susceptible patient into the model using the evolutionary game method. We also assume the cure rate and mortality are time-varying which is more reasonable in a realistic situation. By extensive simulation, we find that the role of *α* has limited. Once *α* is higher than certain values, it has little impact on the cumulative number of confirmed cases and death. The effect of *α* depends on the rate to go to the hospital. When the system is normal, the *m* play a positive role. However, facing the pandemic with a high infectious rate, *m* is a two-edged sword. A low level of *m* can contain the pandemic, but a high level makes the pandemic worse. To verify our model, we use real data to obtain the parameter estimates that can fit the model well. Through simulations, the number of medical resources is also assessed. The results show that Japan still has room to improve its medical resources to contain the pandemic efficiently. The findings bring a perspective to understanding the relationship between the transmission of epidemic and human behaviors. Our model provides an available tool to assess whether medical resources are adequate when facing a pandemic.

Pandemic containment depends on medical resources, government measures, human behaviors, and many other factors [54–58]. When an outbreak occurs, the breakdown of the health system will indirectly accelerate the outbreak epidemic. Therefore, how to evaluate the level of the medical system quickly and accurately can not only ensure the sufficiency of medical resources but also avoid excessive waste of resources. We only list the lines graph in studying the evolutionary dynamics of the pandemic, the phase graph also can be calculated similarly for involved parameters but is omitted in this paper since the lines graphs are enough to study the problems we are interested in. In addition, we perform a sensitivity analysis with respect to model parameters in the above results.

## Supporting information

S1 Data (XLSX)

S1 File (PDF)
