# Supplementary material for: Assessing the medical resources in COVID-19 based on evolutionary game
Source: PLoS One. 2023 Jan 11;18(1):e0280067. doi: 10.1371/journal.pone.0280067 (PMC9833555; doi:10.1371/journal.pone.0280067)
Supplement: S1 File — (PDF) [file pone.0280067.s002.pdf]

Data source and preprocessing

We gathered epidemiological data from the following publicly available data sources:

WHO (https://covid19.who.int/) and

The Ministry of Health (https://www.worldometers.info/coronavirus/).

We can observe the death toll, the number of recoveries and newly infected for each day, and the sum of indicators is the cumulative number of diagnoses. With the aid of the indicator 'the cumulative number of diagnoses', we can evaluate the disease severity. For the purpose of assuring data reliability, the same values which are from the above websites are retained; otherwise, the values taken from data published by the governments. Sampling time shows up in Fig. 5

Algorithm

To find the estimation of parameters in an epidemic model, several methods have been suggested such as least square method [56], gradient descent [57], grid search algorithm [42], and so on. Due to the complexity of the model suggested here, we find the grid search algorithm performs well compared with other methods. The idea of the grid search algorithm is to find the best parameter estimators by evaluating the efficiency of all possible combinations of parameters over grid space. In the grid search algorithm as has been proposed, we use mean square error as a criterion for evaluating the model

$$MSE = \frac{1}{n}\sum_{t=1}^{n}\sum_{i=1}^{2}(\hat{y}_{it} - y_{it})^2,$$

where y_{it} is the cumulative number of deaths for $i = 1$ and illnesses for $i = 2$ at time t, $\hat{y}_{it}$ is the predicted value, and n is the number of the time period in the early stage of pandemic.

The algorithm of grid search (see algorithm 1) optimizer used model in this study can be summarized below:

In the first step, we set the initial condition of models where the total population comes from WHO of 2019, and initially infected seeds are chosen at the start of the pandemic. At the same time, a threshold ψ whose value is 0.001, is given to measure the fitting precision of the model. Thus, initial state $S, E, I, A, H, D, R, \delta_2$ and μ_2 need to be given. Note that initial values of δ_2 are $\delta_{max}$and μ_2 is δ_{min} The parameters involved in the model (2) range from 0 to 1, thus we grid the [0,1] interval with a span of 0.1 for each parameter, and finally form a parameter set $B = [\beta, \theta, \epsilon, \sigma, \alpha, r, s, m]$, which contain all possible combinations of hyperparameters over the grid set. In the second step, we solve equations (2) and find the best combination of hyperparameters τ that makes MSE attain a minimum. If $MSE \leq \psi$ for this value τ, the algorithm is

Algorithm 1 grid search algorithm

Input: Cumulative number of deaths y_{1t}, cumulative number of illnesses y_{2t}, error threshold ψ, initial state $S, E, I, A, H, D, R, \delta_2, \mu_2$ and parameter set $B = [\beta, \theta, \epsilon, \sigma, \alpha, r, s, m]$

Output: The best parameter set τ

1: listing all possible combinations of hyperparameters about parameter set B, and every combination of hyperparameters is regarded as a grid point.
2: Predictive value solution: solving equations (2) by introducing the grid point, which we can get $\hat{y}_{1t}$ and $\hat{y}_{2t}$. Namely, $\hat{y}_{1t}$ and $\hat{y}_{2t}$ are gotten by solving equations (2) with every grid point.
3: solving MSE for each grid point.
4: **if** MSE $\geq \psi$ **then**
5: seeking the best combination of hyperparameters τ at step two which can minimize MSE
6: **Parameter set refinement:** range of parameters B become form $\tau - \Delta$ to $\tau + \Delta$, and $\Delta = 0.1\Delta$
7: **else**
8: the chosen parameter set τ produces.
9: **return** τ

terminated, otherwise go to the third step. In the third step, the hyperparameters produced in the second step further be gridded. That is to say, the range of every parameter for searching becomes $\tau - \Delta$ to $\tau + \Delta$, and the span interval of the grid is set by $\tilde{\Delta} = 0.1\Delta$. The grid search processing is then similarly conducted as that in the second step. This procedure is repeated until the MSE is less than ψ.

Robust Analysis

Diverse behavior patterns induce different probabilities of going to the hospital. In the probabilistic functions characterizing behavioral patterns, the maximum effect [29], conformity [30,31] and fixed values [23,24,28,49,58] are common. In the results section, we present the results by function with the maximum effect. Here, we consider other behavior patterns: functions with behavioral (conformity-driven update rule) and without behavioral. We define the conformity-driven update rule as follows

$$P(H(t), I(t), A(t)) = \frac{m}{1 + exp((H(t)/N - (I(t) + A(t))/N)/K)}, \tag{S1}$$

This rule is reflective of conditions where individuals choose to hospital and learn about those who don't go to the hospital. When the ratio of being tested and being sent to the hospital is greater than those the fraction of symptomatic individuals and asymptomatic individuals, symptomatic individuals tend more to go to the hospital.

Given rules without behavior, P is a constant and can be calculated by the grid search algorithm ($P = 0.3$).

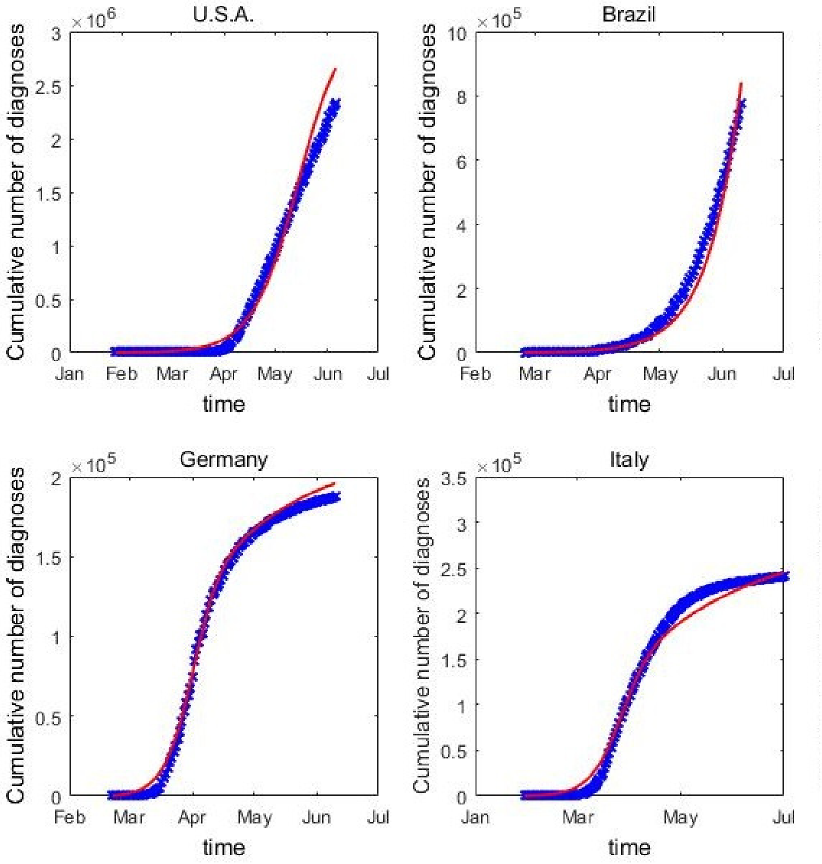

Fig S1. The solid lines are obtained from the theoretical model, and the dots correspond to the real cases. These countries contain the United States, Brazil, Germany, and Italy. The parameters in this model are selected from the fitting results in Table S1.

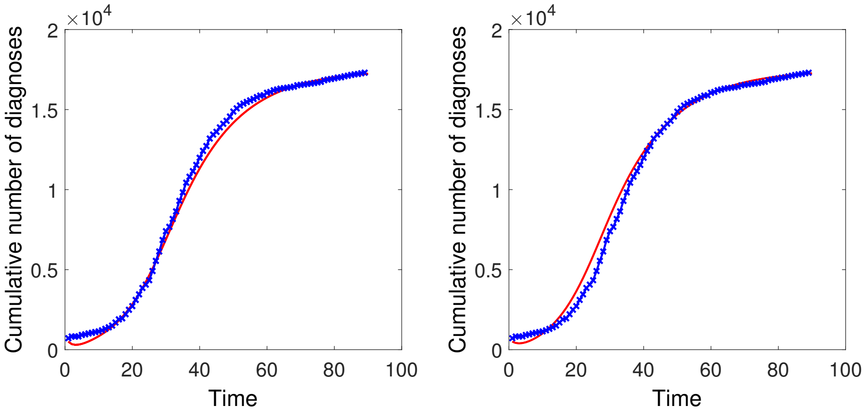

Fig S2. The solid lines are obtained from the theoretical model, and the dots correspond to the real cases. From left to the right, update rules are conformity-driven update rules and patterns without behavioral. The parameters in this model are selected from the fitting results in Table 3.

Table S1. Parameter estimation fitting the data from four countries

Symbol	U.S.A.	Brazil	Germany	Italy
μ_{max}	0.02	0.06	0.17	0.13
μ_{min}	1e-4	0.001	0.02	0.01
μ_1	0.01	0.04	0.02	0.012
δ_{max}	0.015	0.06	0.007	0.004
δ_{min}	3e-4	0.02	9e-4	1.5e-3
δ_1	1.5e-3	0.02	9e-4	0.003
α	0.03	0.03	0.2	0.1
β	1	1	1	1
q	0.2	0.214	0.38	0.5
m	0.11	0.12	0.26	0.35
r	2.1	0.08	4e-4	4e-4
s	2.9	0.09	3e-4	3e-4
ϵ	0.007	0.07	2.8e-4	5.1e-3
σ	0.18	0.2	0.6	0.15
θ	1e-4	1e-5	1e-5	1e-5
Initial Data	Jan. 23th	Feb. 25th	Jan. 28th	Jan. 30th
